# Anti-Inflammatory Effect of Fermented Cabbage Extract Containing Nitric Oxide Metabolites with Silica

**DOI:** 10.3390/ijms25020775

**Published:** 2024-01-08

**Authors:** Yun-Seong Lee, Byeong-Jun Ji, Hyun-Ock Pae, Mu-Weon Cheon, Guangpeng Xu, Hyun-Soo Chun, Sooah Kim

**Affiliations:** 1 Department of Microbiology and Immunology, School of Medicine, Wonkwang University, Iksan 54538, Republic of Korea; iyoonseong@daum.net (Y.-S.L.); hopae@wku.ac.kr (H.-O.P.); 2 HumanEnos LLC., Wanju 55347, Republic of Korea; wandukong5@naver.com; 3 Department of Biomedical Sciences and Institute for Medical Science, Chonbuk National University Medical School, Jeonju 54907, Republic of Korea; 4 Department of Chemistry, KwangWoon University, Seoul 01897, Republic of Korea; cmw10000@naver.com; 5 Department of Environment Science & Biotechnology, Jeonju University, Jeonju 55069, Republic of Korea; xgp947@gmail.com

**Keywords:** atopic dermatitis, fermented cabbage extract, nitric oxide metabolites, silica, anti-inflammatory

## Abstract

The aim of this study was to evaluate the anti-inflammatory effect of fermented cabbage extract (FC) containing nitric oxide metabolites with silica (FCS) on 1-fluoro-2,4-dinitrofluorobenzene (DNFB)-induced atopic dermatitis (AD) in BALB/c mice. Atopic dermatitis-like allergic contact dermatitis was induced by DNFB challenge in the ear after DNFB sensitization on the dorsal skin of mice. FCS alleviated the severity of atopic dermatitis-like skin lesions. In addition, epidermis thickness of the ear and penetration of inflammatory cells in atopic dermatitis-like skin lesions were decreased after topical application of FCS. The serum levels of TNF-α and IL-4 were measured in atopic dermatitis mice using ELISA kits, which were observed to be significantly decreased after topical application of FCS. This study demonstrates that the FCS can be used as a potential therapeutic for the treatment and prevention of AD.

## 1. Introduction

Atopic dermatitis (AD), also known as atopic eczema, is a chronic inflammatory skin disease characterized by dry skin that damages the skin barrier due to increased transepidermal water loss [1]. The stratum corneum, which is the outermost part of the epidermis, acts as the skin barrier; its dysfunction results in increased skin permeability of irritating substances, causing exacerbation of inflammation, which further accelerates the skin barrier damage [2,3]. In AD, the levels of inflammatory cytokines, including tumor necrosis factor alpha (TNF-α), are increased through the induced expression of intercellular adhesion molecule-1 (ICAM-1), which is a prerequisite for lymphocyte infiltration into the epidermis to initiate eczematous reaction [4]. Exposure to dinitrofluorobenzene (DNFB) induces contact dermatitis in mice [5]. When the shaved dorsal skin of mice was exposed to DNFB, cytotoxic T lymphocytes were recruited to the skin, resulting in keratinocyte apoptosis and skin barrier dysfunction [6]. Therefore, there is an increasing demand for safe pharmaceutical alternatives to steroids and antihistamines.

Nitric oxide (NO) plays an important role in immune function and vasodilation and is thus involved in maintaining skin homeostasis [7]. Recent research has demonstrated that polymers that can continuously release low levels of NO greatly reduce the platelet activation and adhesion on the surfaces of various materials in in vivo tests [8]. Nitric oxide is well known as a potent antiplatelet agent, and its continuous release from the surface of endothelial cells effectively prevents the activation of platelets on the walls of healthy blood vessels [9]. The NO-flux from normal and stimulated endothelial cells has been estimated to be in the range (0.5–4.1) × 10^−10^ mol/cm^2^·min [10,11]. Also, NO metabolites, including nitrite (NO_2_^−^) and nitrate (NO_3_^−^), usually evaluated together and expressed as NOx, may play a positive role in stable clinical conditions, because they can be reduced to NO once again [12]. 

Silica particles have also been used as precursors for the preparation of NO-releasing agents. Meyerhoff’s group reported [8] the first example of the use of NOate-modified fumed silica as NO-releasing agents in 2003. The same group reported the SNO-modified NO-releasing fume silica particles in 2005 [13]. In skin tissue, silica nanoparticles can release silicon ions that aid in angiogenesis and collagen deposition during the healing of wounds by activating the hypoxia-inducible factor (HIF)-1α and vascular endothelial growth factor (VEGF) signaling pathways [14,15]. Moreover, silica nanoparticles exert nanobridging effects on soft tissue matrices that accelerate wound closure [16,17]. In particular, previous studies have shown NO-releasing nanoparticles as potential treatment options for skin diseases such as AD and ischemic wounds [18,19]. 

Cabbage production has recently received increased attention because of its anticancer, antifibrotic, anti-inflammatory, anti-diabetic, and antioxidant activities [20,21,22,23]. Cabbage alleviates diseases associated with oxidative stress by scavenging reactive oxygen and nitrogen species, decreasing lipid peroxidation, stimulating the generation of antioxidant cytoprotective proteins, and suppressing inflammatory cytokines [24,25,26]. It can also chelate oxidative metal ions such as lead, preventing their accumulation in various tissues; moreover, it leads to improved hematological parameters, alleviation of inflammation, and protection and repair of cells [27]. The fermentation process can improve the biological properties of the starting ingredients by altering the biochemical composition [28]. Previous studies have reported that fermented foods protect against AD [29,30]. 

**Figure 1:**
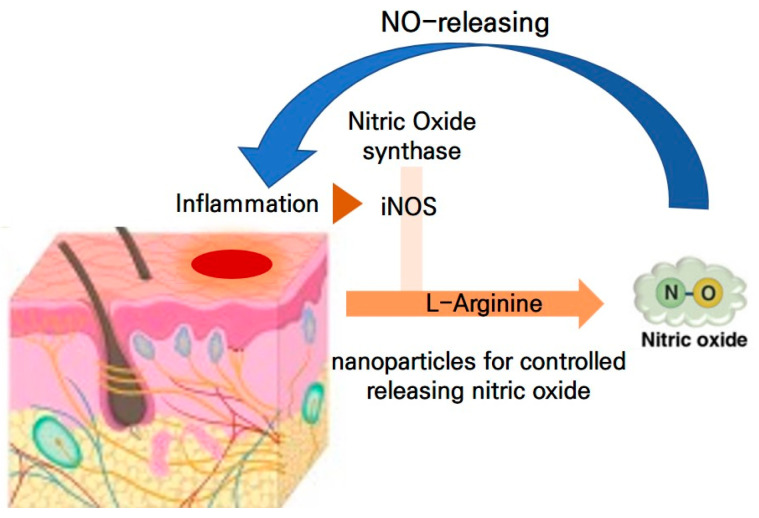
NO-releasing biomaterials in the treatment of skin inflammation.

However, to the best of our knowledge, few studies have investigated the effect of fermented cabbage extract containing NO metabolite-releasing nanoparticles against AD, despite their potential health effects. Therefore, in this study, we used 4-dinitrofluorobenzene (DNFB)-induced AD in mice to evaluate the anti-inflammation effects of fermented cabbage extract containing NO-releasing nanoparticles against AD (Figure 1).

## 2. Results

### 2.1. Determination of Fermented Cabbage Extract Containing Nitric Oxide 

**Figure 2:**
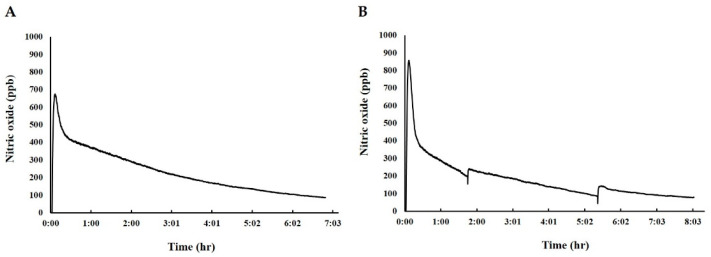
Representative line histograms showing time-dependent changes in NO release from (**A**) fermented cabbage extract (FC) and (**B**) FCS. Panels (**A**,**B**) show the concentration (ppb) of NO over time.

NO concentration was the highest at 6 min in both fermented cabbage extract (FC) and fermented cabbage extract with silica (FCS), and it was 674.4 ppb (Figure 2A) and 857.4 ppb (Figure 2B), respectively, with FCS containing higher content of NO than that of FC. In addition, the duration of NO release in FCS was maintained for 1 h longer than that in FC. 

### 2.2. GABA and Total Polyphenol Content of Fermented Cabbage Extract with Silica (FCS)

**Figure 3:**
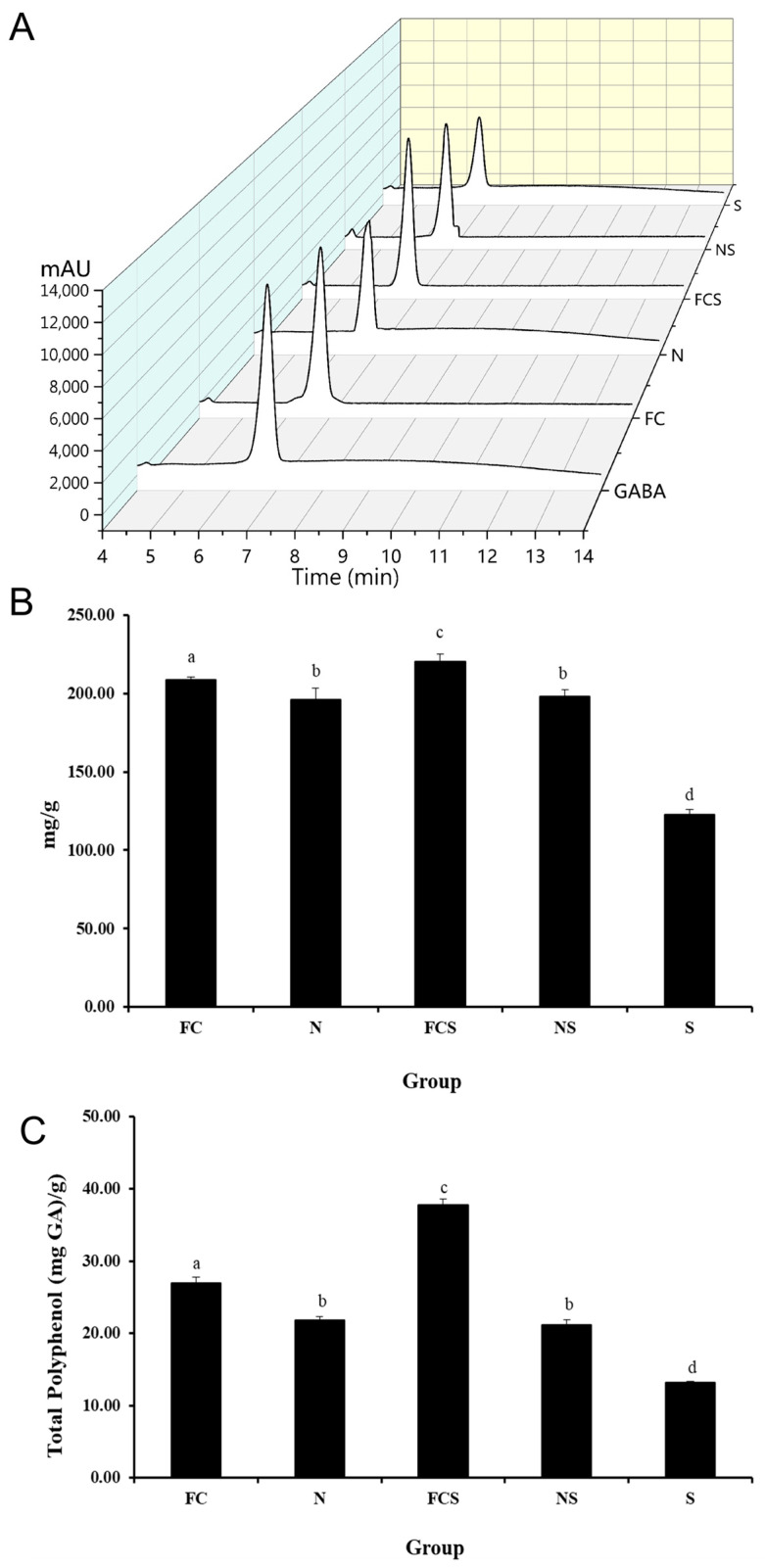
(**A**) HPLC chromatogram of GABA of standard, cabbage extract (FC), sodium nitrate (N), fermented cabbage extract with silica (FCS), sodium nitrated with silica (NS), and silica (S). The chromatograms were generated by using OriginPro 2021b software. Content of (**B**) GABA and (**C**) total polyphenols from the samples. GABA and total polyphenol levels were measured by HPLC and Folin–Ciocalteu method, respectively. Results are represented as the mean ± SE. ^a–d^ Means with different superscripts are significantly different (*p* < 0.05) as analyzed using Duncan’s multiple range test.

The previous studies reported that γ-aminobutyric acid (GABA) and polyphenol have a potential effect to alleviate AD [31,32]. Therefore, we compared GABA and total polyphenol contents of fermented cabbage extract (FC), sodium nitrate (N), fermented cabbage extract with silica (FCS), sodium nitrated with silica (NS), and silica (S) (Figure 3). As shown in Figure 2A, the mean elution time for GABA is approximately 6.5 min at 360 nm (Figure 3A). The GABA peaks of FC, N, FCS, NS, and S were detected, and the GABA concentration of the samples was calculated using the calibration curve (Figure 3A). The GABA level of FCS (208.80 ± 1.79 mg/g) was 1.8 times higher than that of S (122.94 ± 3.21 mg/g) (Figure 3B). In addition, the GABA level of FCS was higher than FC (208.80 ± 1.79 mg/g), N (196.09 ± 7.19 mg/g), and NS (198.22 ± 4.03 mg/g). The level of total polyphenol content of FCS was much higher than that of FC, N, NS, and S (Figure 3C). These results indicated that the FCS could improve the anti-inflammatory effect of AD. 

### 2.3. Fermented Cabbage Extract with Silica (FCS) Treatment Reduced AD Severity in DNFB-Induced Atopic Dermatitis Mice

**Figure 4:**
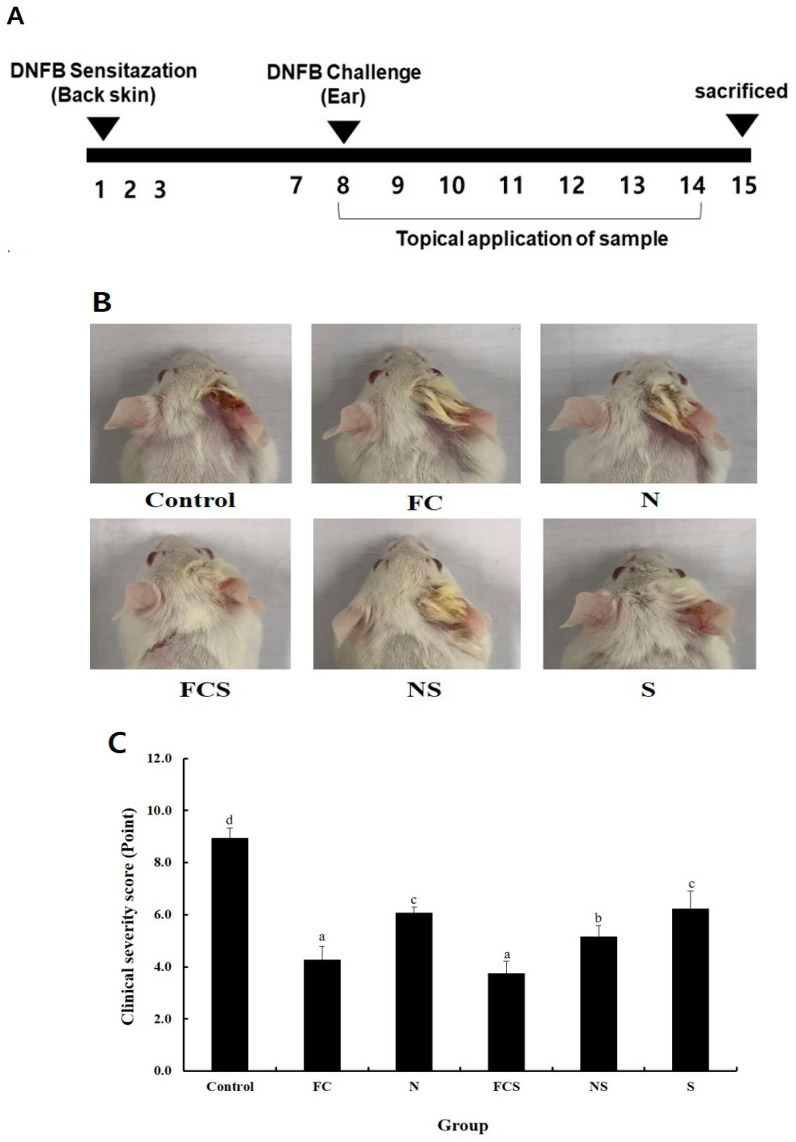

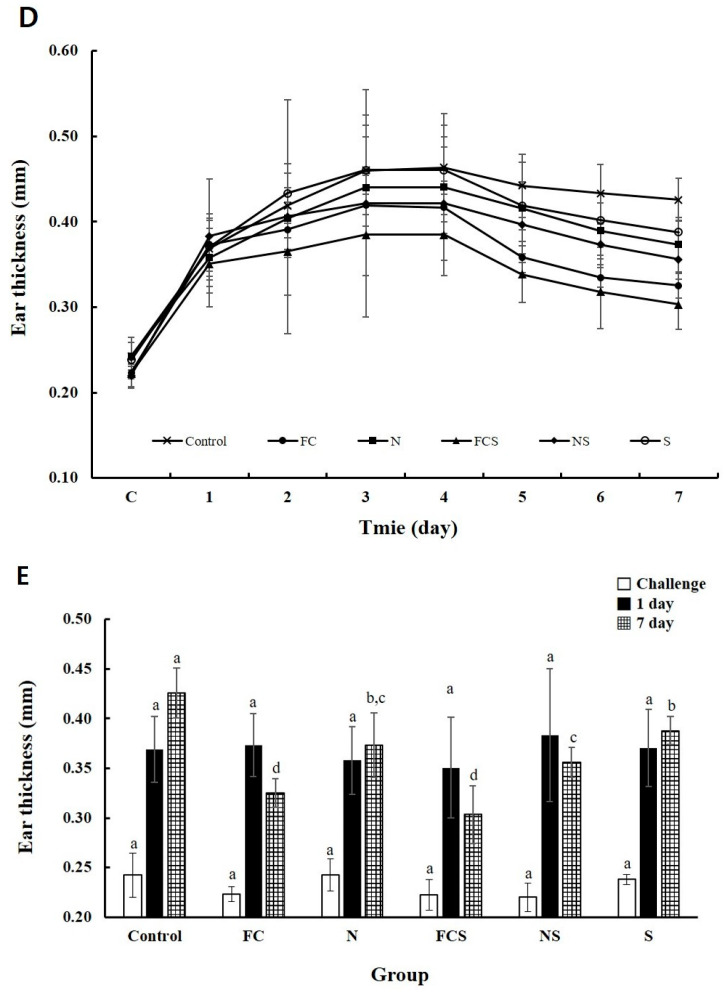
Fermented cabbage extract with silica (FCS) attenuated symptoms in DNFB-induced AD mice. (**A**) Experimental design. Mice in the experimental groups were sensitized by applying 0.5% DNFB on days 1, 2, and 3. The mice were then challenged by 0.3% DNFB on day 14. Group was topically applied with FCS (20 μL) for seven days. (**B**) BALB/c mice developed chronic skin inflammation, and healing activity was initiated. (**C**) Clinical severity score. (**D**) Changes in ear epidermis thickness. (**E**) Ear thickness was measured on day 7. Control, 0.3% DNFB + vehicle; FC, 0.3% DNFB + FC 200 mg/kg; N, 0.3% DNFB + sodium nitrite 200 mg/kg; FCS, 0.3% DNFB + 0.1% FCS 200 mg/kg; NS, 0.3% DNFB + sodium nitrite with 0.1% silica 200 mg/kg; S, 0.3% DNFB + 0.1% silica 200 mg/kg. Results are represented as the mean ± SE. ^a–d^ Means with different superscripts are significantly different (*p* < 0.05) as analyzed using Duncan’s multiple range test. AD, atopic dermatitis; DNFB, 1-fluoro-2,4-dinitrofluorobenzene; FC, fermented cabbage extract; N, sodium nitrate; FCS, fermented cabbage extract with silica; NS, sodium nitrated with silica; S, silica.

To investigate the effect of fermented cabbage extract with silica (FCS) on AD, FCS was applied to the right ear of DNFB-induced AD mice, and the lesions were visually evaluated after the application period (Figure 4B). FCS and fermented cabbage extract (FC) application improved skin lesions such as dryness and swelling. The clinical severity score of the right ear in DNFB-induced AD mice was 9.0 ± 0.4 in the mice of the control group (control), 4.3 ± 0.5 in the mice treated with FC, 6.1 ± 0.2 in the mice treated with sodium nitrate (N), 3.8 ± 0.5 in the mice treated with FCS, 5.2 ± 0.4 in the mice of sodium nitrated with silica (NS), and 6.2 ± 0.7 in the mice treated with silica (S) groups (Figure 4C). In fermented cabbage extract (FC) and fermented cabbage extract with silica (FCS) treatment groups, the dermatitis score was much lower than that in the control groups. In all experimental groups, the symptoms of atopic lesions were significantly reduced compared with those in the control group (distilled water) (*p* < 0.05). The treatment of fermented cabbage extract (FC) and fermented cabbage extract with silica (FCS) showed a significant decrease in ear thickness compared to control after 7 days (control: 0.43 ± 0.03 mm, FC: 0.33 ± 0.01 mm, N: 0.37 ± 0.03 mm, FCS: 0.30 ± 0.03 mm, NS: 0.36 ± 0.02 mm, and S: 0.39 ± 0.01 mm) (Figure 4D,E). These results indicated that the FCS can be used as a potential treatment by alleviating the symptoms of AD.

### 2.4. Effect of Fermented Cabbage Extract with Silica (FCS) to Cytokine and Spleen Index in DNFB-Induced AD Mice

**Figure 5:**
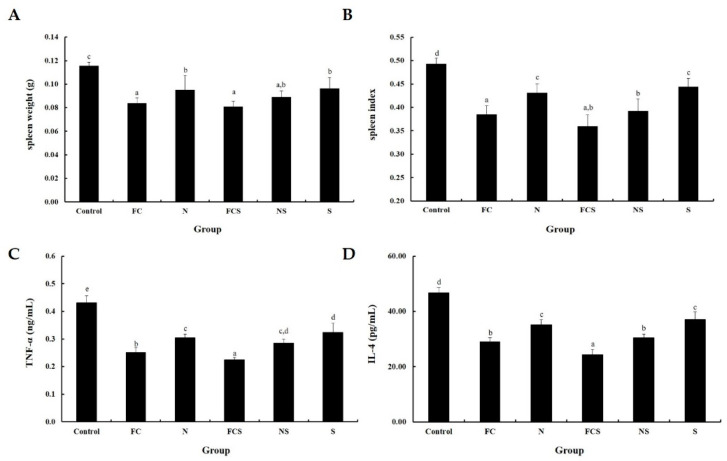
Effect of FCS in DNFB-induced AD mice. (**A**) Spleen weight and (**B**) spleen index. Serum level of inflammatory cytokines (**C**) TNF-α and (**D**) IL-4. Control, 0.3% DNFB + vehicle; FC, 0.3% DNFB + FC 200 mg/kg; N, 0.3% DNFB + sodium nitrite 200 mg/kg; FCS, 0.3% DNFB + 0.1% FCS 200 mg/kg; NS, 0.3% DNFB + sodium nitrite with 0.1% silica 200 mg/kg; S, 0.3% DNFB + 0.1% silica 200 mg/kg. Results are represented as the mean ± SE. ^a–e^ Means with different superscripts are significantly different (*p* < 0.05) as analyzed using Duncan’s multiple range test. AD, atopic dermatitis; DNFB, 1-fluoro-2,4-dinitrofluorobenzene; FC, fermented cabbage extract; N, sodium nitrate; FCS, fermented cabbage extract with silica; NS, sodium nitrated with silica; S, silica.

To investigate whether fermented cabbage extract with silica (FCS) decreases the spleen, the spleen indices of DNFB-induced AD mice were calculated (Figure 5A,B). The results showed the spleen weight and index decreased by FCS application. We further examined the expression of serum cytokines to determine the effect of FCS on inflammatory responses in DNFB-induced AD mice. ELISA results indicate that TNF-α and IL-4 levels were higher in DNFB-induced AD mice than those in mice of the control group (Figure 5C,D). The results showed that the application of FCS can suppress the expression of serum cytokines, indicating that FCS downregulates the immune responses. 

### 2.5. Histological Analysis from Ear Tissue of DNFB-Induced AD Mice

**Figure 6:**
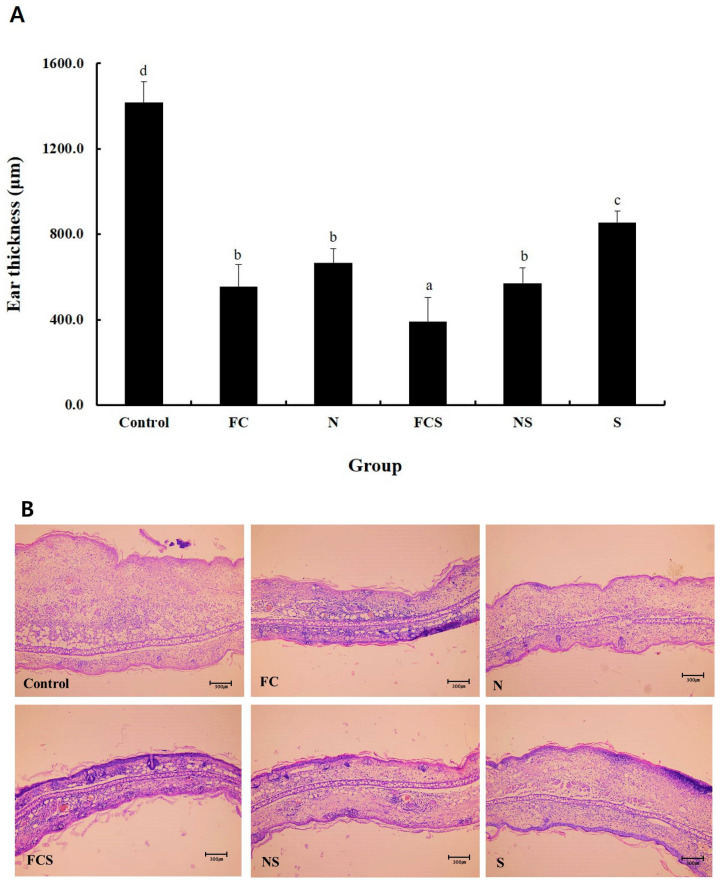
Histochemical analysis of (**A**) ear thickness and (**B**) skin epidermis via H&E staining (×100). Control, 0.3% DNFB + vehicle; FC, 0.3% DNFB + FC 200 mg/kg; N, 0.3% DNFB + sodium nitrite 200 mg/kg; FCS, 0.3% DNFB + 0.1% FCS 200 mg/kg; NS, 0.3% DNFB + sodium nitrite with 0.1% silica 200 mg/kg; S, 0.3% DNFB + 0.1% silica 200 mg/kg. Results are represented as the mean ± SE. ^a–d^ Means with different superscripts are significantly different (*p* < 0.05) as analyzed using Duncan’s multiple range test. DNFB, 1-fluoro-2,4-dinitrofluorobenzene; FC, fermented cabbage extract; N, sodium nitrate; FCS, fermented cabbage extract with silica; NS, sodium nitrated with silica; S, silica.

The ear epidermal tissues of BALB/c mice were stained with H&E and examined using an optical microscope. The relative change in ear epidermal tissue thickness was fermented cabbage extract (FC) 9.1 ± 7.3%, sodium nitrate (N) 47.0 ± 4.7%, fermented cabbage extract with silica (FCS) 27.7 ± 7.9%, sodium nitrated with silica (NS) 40.3 ± 4.9%, and silica (S) 60.4 ± 3.7%, compared with the mean value of the control group set at 100% (Figure 6A). The measured thickness of the epidermal tissues of the right ear was control 1417.9 ± 96.7 μm, FC 554.3 ± 103.9 μm, N 666.1 ± 67.0 μm, FCS 392.6 ± 111.3 μm, NS 572.1 ± 70.1 μm, and S 856.0 ± 52.2 μm (Figure 6B). All experimental groups showed significant differences compared to the control group (*p* < 0.05). AD is a representative allergic inflammatory skin disease. Patients with allergic inflammatory skin diseases typically exhibit symptoms such as keratinization, swelling, and erythema [33]. Inflammatory hypersensitivity reactions promote the differentiation of T cells and the release of various inflammatory cytokines, including IL-4, by Th2 cells [34]. Excessive IL-4 secretion degranulates immune cells, exacerbates epidermal barrier dysfunction, and causes itching [35,36]. FCS improved AD-like lesions and severe scratching behavior, IL-4 was decreased, and ear thickness was induced by DNFB. In the histological study, H&E staining showed FCS significantly reduced mast cell infiltration and epidermal thickening. In addition, in FCS treated group, the epidermal thickness significantly decreased compared to other groups, indicating the application of FCS was the most effective for the treatment of AD. 

## 3. Discussion

In our study, the beneficial effects of FCS in the DNFB-induced AD mice model were investigated. AD is a chronic inflammatory skin disease characterized by pruritic eczema and scratching, which can induce skin injury [37]. For many years, AD has been thought to be the first manifestation of atopy (the familial propensity to become IgE-sensitized to environmental allergens) and the initial step in the so-called atopic march, which ultimately leads to asthma and allergic rhinitis [38]. Therefore, research into pathogenesis, prevention, and treatment focused on sirolimus, cyclosporine A, and dexamethasone, which are clinically relevant immunosuppressive drugs with distinct molecular mechanisms of action and different immunoregulatory profiles. The effects of these drugs on dendritic cell differentiation, migration, and maturation have been previously reported [39]. Recent research has highlighted the antimicrobial properties of NO [40,41], a reactive free radical produced by inflammatory cells (neutrophils and macrophages) to combat infection. Using small-molecule NO donors, Raulli et al. demonstrated that NO possessed antibacterial properties against a wide range of Gram-positive and Gram-negative bacteria [42]. NO is a versatile molecule that plays a role in various biological processes, including immune defense, inflammation, and neurotransmission [43]. NO is a short-lived molecule (with a half-life of 6 s) produced by enzymes known as nitric oxide synthases (NOSs) and acts as an L-arginine substrate and is transported into cells [44]. Being a small molecule, NO can penetrate rapidly across cell membranes and diffuse through distances greater than several microns. Since NO can be formed or synthesized in various tissues, it can affect several important biological processes and has been implicated in several diseases [45]. NO is often expressed in the skin in several instances. During Leishmania infection, NO production is pivotal for the inhibition and destruction of Leishmania parasites in the cutaneous lesions and draining lymph nodes of resistant mice [46]. Keratinocyte-derived NO production accompanies wound healing [47]. Inducible nitric oxide synthase (iNOS) expression has been observed in the skin lesions of patients with psoriasis [48]. NO is involved in the maintenance of resting vascular tone in human skin and causes vasodilation in response to local warming or UV B irradiation [49]. As an alternative strategy for delivering NO to pathogenic bacteria, recent studies have reported antibacterial properties of NO-releasing silica nanoparticles. The nanoparticles exhibited enhanced bactericidal efficacy against planktonic Pseudomonas aeruginosa cells compared with small-molecule NO donors [50]. However, the effectiveness of NO-releasing nanoparticles against established biofilms remains unclear. The rapid diffusion property of NO may result in its enhanced penetration into the biofilm matrix, thus improving its efficacy against biofilm-embedded bacteria [51,52]. Moreover, a promising advantage of nanoparticles over small molecules is that their physicochemical properties (e.g., hydrophobicity, charge, and size) can be tuned by varying the synthetic precursors and procedures [53,54]. Regarding health-promoting properties, as fermented cabbage is rich in antioxidants such as vitamin C and ascorbigen (ABG) [55], it has a high antioxidant potential [56]. Fermented cabbage contains ABG, which is formed by the enzymatic hydrolysis of glucobrassicin to indol-3-carbinol (I3C), and its subsequent reaction with L-ascorbic acid [57]. Moreover, glucosinolates hydrolysis products in sauerkraut, such as ABG, I3C, sulforaphane (SF), allyl isothiocyanate (AITC), butyl isothiocyanate (BITC), and phenylethyl isothiocyanate (PITC) [58,59,60], have been shown to be effective in attenuating oxidative stress by upregulating the expression of antioxidants and phase 2 enzymes [61,62]. Fermented cabbage extracts containing NO metabolites with silica increased NO emissions and release time. This suggests that NO increases the concentration and time required to respond to inflammatory sites. Inflammation increases blood flow, resulting in fever and skin flares. Neutrophils in the blood permeate out of blood vessels, causing edema and activating cytokines at the site of inflammation [63]. TNF-α is a pro-inflammatory cytokine responsible for the initiation of an inflammatory response and acts alone or in collaboration with Th2 cytokine to regulate the lipid barrier function of AD skin. TNF-α activates immune cells such as macrophages and neutrophils, and especially, IL-4, and boosts immunity [64,65,66]. The experiment was conducted based on the findings of previous studies that damage to the experimental skin barrier increases the secretion of Th2 cytokines such as IL-4 and IL-13, causing an inflammatory reaction represented by an immunological condition of Th2 cytokine dominance [67,68], and studies that reported an increase in the levels of the cytokines IL-1α and TNF-α [69]. TNF-α and IL-4 levels were decreased in the FCS group compared to the FC group. Thus, these findings indicate control of Th2 differentiation by alleviating damage to the FCS skin barrier and contributing to alleviating skin damage by inhibiting the expression of inflammatory mediators such as TNF-α and IL-4. In addition, it was confirmed to be an effective substance for atopic dermatitis by suppressing neutrophil infiltration through pathological observations and inhibiting the secretion of inflammatory cytokines in the blood. Conclusively, liquid FCS is considered a potential substance for the prevention and treatment of AD.

## 4. Materials and Methods

### 4.1. Preparation of Fermented Cabbage Extract

The study used fermented cabbage extract (FC) manufactured by Human Enos (Wanju-gun, Korea) [70]. Ground cabbage was mixed with distilled water in a 1:1 ratio, and then approximately 1% (1.0 × 10^8^ cfu/mL) of generally recognized as safe (GRAS)-grade microorganisms were added. Fermentation was performed at 30 °C for 21 days under controlled optimal conditions of aeration, temperature, and pH. The liquid product obtained at the end of 21 days contained NO metabolites and antioxidants. The supernatant was separated using a centrifuge (1.5 ton/h, disc separator; Alfatechkorea Corp., Hwaseong-si, Gyeonggi-do, Republic of Korea) and then condensed using an evaporator (1.5 ton, vacuum evaporator; BDMPLANT, Gwangju-si, Gyeonggi-do, Republic of Korea), yielding a Brix of 4%. The condensed material was then frozen at –40 °C for 48 h and placed in a freeze dryer (1.5 ton, vacuum freeze drier; Ajin E.S.R Co. Ltd., Daegu, Republic of Korea) for 72 h to obtain the powder form. Nitrite levels in FC were determined using the Griess reagent (Promega, Madison, WI, USA) according to the manufacturer’s protocol. The fermented cabbage extracts (FC) used in this study were fermented for 21 days. Fermented cabbage extract with silica (FCS) was prepared by mixing FC with 0.1% silica that was purchased from CEN Co., Ltd. (Miryang-si, Republic of Korea; http://cen2701.com/ (accessed on 25 May 2022)).

### 4.2. GABA and Total Polyphenol Content

The GABA continent was analyzed using method described previously [71,72]. Briefly, the samples, including cabbage extract (FC), sodium nitrate (N), fermented cabbage extract with silica (FCS), sodium nitrated with silica (NS), and silica (S) were mixed with 1.5 mL of 0.5 M sodium bicarbonate and 0.5 mL of 0.715 mg/mL 1-fluoro-2,4-dinitrobenzene. The mixture was incubated at 60 °C for 1 h and then centrifuged at 12,000 rpm for 5 min at 4 °C. The supernatant was filtered through a 0. 22 μm syringe filter (Hydrophilic PTFE; Advantec, Dublin, OH, USA). High-performance liquid chromatography (HPLC) analysis was performed with an LC-20AD pump, an SPE-M20A diode array detector, a CTO-20A oven, a CBM-20A con-troller, and an SIL-20A autosampler (HPLC; Simazhu, Kyoto, Japan). The separation was carried out on a Symmetry C18 column (3.9 × 150 mm, 5 μm), thermostated at 30 °C, using 0.5% ammonium acetate aqueous solution and acetonitrile (85:15, *v*/*v*) as the mobile phase a flow rate of 1 mL/min for 20 min. The UV/Vis detector was performed at 360 nm to measure concentration of GABA. The Folin–Ciocalteu method was used for determining total polyphenol content [73]. Briefly, 16 μL of samples such as FC, N, FCS, NS, and S solution was mixed with 60 μL of Folin–Ciocalteu reagent. After incubation of the mixture at 25 °C for 5 min, 60 μL of 129 g/L sodium carbonate solution was added and placed in a dark place for 90 min. The absorbance was measured at 725 nm using a UV spectrophotometer (UV-1800, Shimadzu, Kyoto, Japan), and the total polyphenol content was represented in mg of gallic acid/g dry weight (mg GA/g dry weight). 

### 4.3. Experimental Animals and Group Design

Five-week-old male BALB/c mice (male) were acquired from Samtako (Osan, Republic of Korea) and used after a one-week acclimation period. The breeding room was maintained at a temperature of 23 ± 2 °C and a humidity of 50–60%, food and water were supplied freely, and the brightness was adjusted as 12 h light/dark cycle. Animal experiments were conducted in accordance with the guidelines for the care and use of experimental animals (approval no. WKU22-132). Atopic dermatitis was induced by applying 0.3% DNFB (1-fluoro-2,4-dinitrobenzene) to the right ear in the BALB/C mice, and atopic dermatitis was not induced in the left ear of each mouse. Experimental animals were divided into four groups according to the samples applied to the right ear: vehicle control group applied distilled water, FC group applied fermented cabbage extracts, N group applied sodium nitrite (NaNO_2_), FCS group applied fermented cabbage extracts with silica 0.1%, NS group applied sodium nitrite (NaNO_2_) with silica 0.1%, and S group applied silica 0.1% (Table 1). AD was induced as previously described. DNFB (1-fluoro-2,4-dinitrobenzene) reagent (Sigma-Aldrich, St. Louis, MO, USA) was prepared by dilution in acetone: olive oil (4:1). After a one-week acclimation period, dorsal skin of BALB/c mice were shaved; mice were allowed to acclimate for another three days. Skin sensitization was induced by applying 50 μL of 0.5% DNFB to the shaved back for three days, and 20 μL of 0.3% DNFB was applied to the right ear after five days of skin sensitization to induce AD. The fermented extract (50 μL) suitable for each group was applied to atopic lesion induced in the right ear twice a day for seven days (Figure 3A).

### 4.4. Clinical Assessment of Atopic Dermatitis

AD symptoms were divided into five categories (erythema, pruritus and dry skin, edema and excoriation, erosion, and lichenification), and the severity of each category was as follows: no symptoms (0 points), weak (1 point), moderate (2 points), and severe (3 points). The scores for each category are summed and rated from a minimum of 0 points (no symptoms in all five categories) to a maximum of 15 points (severe symptoms in all five categories). 

### 4.5. Measurement of Ear Thickness

The thickness of the left and right ears of BALB/c mice with induced AD in the right ear were measured using vernier calipers (Mitutoyo Co., Kanagawa, Japan). The thickness of the left ear, which was set as normal, and the thickness of the right ear, in which AD was induced, were measured and compared for seven days.

### 4.6. Spleen Index

For calculation of the spleen index, BALB/c mice were sacrificed after the sample application period, and weight of the spleen and body of mice were measured. The spleen index was calculated using the following formula:

Spleen index = Spleen weight (g)/Body weight (g) × 100

### 4.7. TNF-α and Interleukin (IL)-4 Levels in Serum

Serum levels of TNF-α and IL-4 were measured using ELISA kits (R&D Systems, Minneapolis, MN, USA). Assay diluent solution (50 μL) and serum (50 μL) were added to the well of the kit and reacted at 25 °C. After 2 h, the wells were washed four times with washing buffer, and 100 μL of mouse TNF-α and IL-4 were added to each well and incubated for 2 h at room temperature. After washing four times with washing buffer, 100 μL of substrate solution was added to each well and reacted at room temperature for 30 min; 100 μL of reaction stop solution was then added, and absorbance was measured at 450 nm using an ELISA reader (SPECTRA max M2; Molecular Devices, San Jose, CA, USA).

### 4.8. Histopathological Examination

Histopathological examination was performed by hematoxylin and eosin (H&E) staining. The ear tissue of BALB/c mice was separated, washed with physiological saline, and removed. The ear tissue was fixed in 10% neutral buffered formalin (NBF), embedded in paraffin, and cut to a thickness of 4 mm to prepare a slide. Ear tissue stained with H&E stain was examined under an optical microscope (Eclipse E200, Nikon, Japan).

### 4.9. Statistical Analysis

The results are presented as the mean ± standard deviation (mean ± SD). Statistical analyses were performed using the SPSS version 12, and regression analysis, correlation analysis, *t*-test, one-way ANOVA, and multiple range test were used as analysis tools. Statistical significance was set at *p* < 0.05.

## 5. Conclusions

Collectively, the results of applying the fermented extract with silica to atopic lesions in the stratum corneum of BALB/c mice showed that edema was significantly reduced in the experimental groups compared to those in the control group. FCS increased NO emissions and NO release time. This also suggests that NO increases the concentration and time required for inflammatory responses. This suggests that recovery progresses rapidly by reducing the secretion of inflammatory cytokines in the inflammatory area.

## Figures and Tables

**Table 1:** Experimental design of animal study investigating the effects of FCS on atopic dermatitis.

Group		Left Ear	Right Ear	Material	Dose	n
1	Control	Normal	0.3% DNFB	Vehicle	-	5
2	FC	Normal	0.3% DNFB	Fermented cabbage extracts	200 mg/kg	5
3	N	Normal	0.3% DNFB	Sodium nitrite (NaNO_2_)	200 mg/kg	5
4	FCS	Normal	0.3% DNFB	Fermented cabbage extracts + silica 0.1%	200 mg/kg	5
5	NS	Normal	0.3% DNFB	Sodium nitrite (NaNO_2_) + silica 0.1%	200 mg/kg	5
6	S	Normal	0.3% DNFB	Silica 0.1%	200 mg/kg	5

## Data Availability

Data are contained within the article.
